# Nuts Improve Diet Quality Compared to Other Energy-Dense Snacks While Maintaining Body Weight

**DOI:** 10.1155/2011/357350

**Published:** 2011-08-10

**Authors:** Siew Ling Tey, Rachel Brown, Andrew Gray, Alexandra Chisholm, Conor Delahunty

**Affiliations:** ^1^Department of Human Nutrition, University of Otago, P.O. Box 56, Dunedin 9054, New Zealand; ^2^Department of Preventive and Social Medicine, University of Otago, P.O. Box 913, Dunedin 9016, New Zealand; ^3^CSIRO Food and Nutritional Sciences, P.O. Box 52, North Ryde, NSW 1670, Australia

## Abstract

Previous studies have reported that regular nut consumption reduces cardiovascular disease (CVD) risk and does not promote weight gain despite the fact that nuts are energy-dense. However, no studies have investigated the body composition of those regularly consuming nuts compared to similar intakes of other snacks of equal energy density. This parallel study (*n* = 118) examined the effects of providing daily portions (~1100 kJ/d) of hazelnuts, chocolate, or potato crisps compared to a control group receiving no snacks for twelve weeks. Effects on body weight and composition, blood lipids and lipoproteins, resting metabolic rate (RMR), appetite indices, and dietary quality were compared. At week 12, there was no significant difference in any of the outcome measurements between the groups except for dietary quality, which improved significantly in the nut group. Nuts can be incorporated into the diet without adversely affecting body weight and can improve diet quality.

## 1. Introduction

Nuts are rich in unsaturated fats, fibre, minerals, vitamins, and phytonutrients [1–5]. Regular consumption of nuts has been associated with reductions in blood cholesterol concentrations and the risk of CVD [6–11]. However, nuts are energy-dense, high-fat foods, meaning that they contain high amounts of energy per unit weight. In general, the consumption of energy-dense foods is associated with weight gain and obesity [12, 13]. Therefore, the public health recommendation to consume nuts on a regular basis could potentially result in weight gain and possibly negate their beneficial effects. Also, the general public perceive nuts as “fattening” and thus might not heed the advice to consume nuts regularly as a means of reducing CVD risk [14]. It is, therefore, important for policy makers to determine whether the regular consumption of nuts may promote weight gain and hence increase obesity rates among the general population and, based on findings, change the wording of the health recommendation to consumers as appropriate to clarify any misperceptions.

Epidemiological studies show that nut consumers tend to be leaner than those who do not regularly consume nuts. This research tends to show an inverse or no association between nut consumption and body mass index (BMI) as well as adiposity [15–19]. Also, clinical trials, where the primary outcomes have included cardiovascular risk factors such as blood cholesterol, have failed to show an increase in body weight with regular consumption of different kinds of nuts, albeit over the short term [7, 9, 20–25]. However, these studies were not designed to assess body weight and in many instances the investigators provided dietary advice or adjusted energy intake to prevent weight gain. 

Studies that have looked at the role of nuts in the context of supervised weight loss diets reported that subjects who consumed nut-enriched diets experienced greater weight loss and greater improvement in CVD risk factors compared with a low-fat diet [26, 27], a complex carbohydrate diet [28], or an isocaloric diet without nuts [29]. Thus, incorporating nuts into diets intended for weight loss and weight control has the potential to result in more favourable changes in body weight and CVD risk. Four randomised crossover trials involving the consumption of nuts have specifically looked at body weight as the primary outcome [30–33]. In general these studies indicate that the daily inclusion of nuts into the diet results in either no weight gain or less weight gain than predicted from the additional energy intake. 

There have been several purported reasons why regular nut consumption may not result in the theoretically predicted weight gain [34–37]. Firstly, nuts are high in protein and fibre with low glycaemic index value, which may promote satiety resulting in a reduction in calories from other foods, that is, dietary compensation [30–33, 38, 39]. The crunchy textural property of whole nuts may also promote satiety as the mechanical act of mastication results in the secretion of appetitive hormones such as cholecystokinin and glucagon-like peptide-1 [28, 40–44]. Secondly, previous work has suggested that nut consumption may lead to an increase in energy expenditure. In humans, a high unsaturated-to-saturated-fat ratio in the diet can increase RMR. Thus, the high unsaturated fat content of nuts may increase RMR [30, 41, 45, 46]. Thirdly, some research has suggested that the lipid found in nuts may not be highly bioaccessible [47, 48], meaning that a high proportion of this fat is excreted in the faeces and therefore not available for energy metabolism [32, 40, 49, 50].

All the above-mentioned studies were not designed to answer the question as to whether nut consumption is different to the ingestion of other isoenergetic foods with regard to dietary compensation and energy metabolism. This information is needed to determine whether there is something unique about nuts, setting them apart from other more highly processed energy-dense foods. It is also needed to confirm current public health advice that nuts, though an energy-dense food, are nutrient-dense and thus should be consumed regularly as part of a cardio-protective diet. Therefore, the purpose of this study was to assess the effects of providing daily portions (~1100 kJ/d) of hazelnuts, chocolate, or, potato crisps for twelve weeks on body weight and composition, blood lipids and lipoproteins, RMR, appetite indices, and diet quality compared with the control group. 

## 2. Materials and Methods

### 2.1. Subjects

One hundred and twenty-four participants were recruited from the general public in Dunedin, New Zealand (NZ). The inclusion criteria were healthy males or females aged between 18 and 65 years. The exclusion criteria were people with BMI ≥ 30 kg/m^2^, people who have asthma, women who are pregnant or breastfeeding, people with a chronic disease such as cancer, heart disease, or diabetes, and people with food allergies or food aversions. The study protocol was approved by the Human Ethics Committee of University of Otago, NZ. All participants gave written informed consent. The trial was registered at the Australian New Zealand Clinical Trials Registry (http://www.anzctr.org.au/), registration number ACTRN12609000265279. 

### 2.2. Test Products

All the Ennis hazelnuts used in this study were purchased from Uncle Joe's Walnuts (Blenheim, Marlborough, NZ). All nuts were stored at room temperature in darkness prior to opening. Dairy milk chocolate (Cadbury, Dunedin, NZ) and ready salted potato crisps (Bluebird, Auckland, NZ) were chosen as comparison foods because they are both popular snack foods in NZ and have a very similar energy density to nuts, with one being savoury and the other sweet. The energy density for the study hazelnuts was 26 kJ/g, while the energy density for the chocolate and potato crisp was 22 kJ/g. 

### 2.3. Study Design

This study was conducted using a randomised, controlled, parallel design with four arms: ~1100 kJ/d for each of hazelnuts (42 g) chocolate (50 g), and potato crisps (50 g) or no additional food (control group). People who were interested in the study contacted the investigator by phone or email. Participants were informed that the purpose of the study was to assess the effects of consuming three different snacks on energy balance and blood cholesterol concentrations. We purposely did not emphasise body weight so that participants were unaware that this was the focus of the research. They were asked to complete a recruitment questionnaire, which included contact information, demographic and relevant health details that might affect the study outcomes. All participants were asked to consume their regular diets (baseline diet) during a two-week run-in period. Baseline measurements were collected from each participant following this run-in period. These included body weight and composition, blood lipids and lipoproteins, RMR, appetite indices, a three-day diet record (3DDR), and physical activity levels. After collecting all baseline measurements, participants were randomly allocated to one of the four groups for a period of 12 weeks.

Due to the strong possibility of age, sex, and BMI effects, groups were balanced using eight strata covering all possible combinations of age group (18–40, 41–65), sex (male, female), and self-reported BMI group (<25, 25–29.9) categories. Allocation within each strata was conducted by an off-site statistician using blocks of size four. Incomplete blocks remaining at the conclusion of enrolment were randomly allocated first using strata based on sex and BMI and then only BMI, and finally without stratification. The statistician was located in another building and had no involvement in the enrolment process. 

All snacks were individually portioned into daily serving sized bags and participants were asked to collect their snacks from the university every three weeks. They received no dietary advice, except that participants in the intervention groups were told that they might consume the snacks however they wished as long as their daily portion was consumed each day. Participants were instructed that the snacks were solely for their own personal consumption and should not be shared with others. They were also asked to return any snacks not eaten on any given day. The control group received no additional food. However, they were given a month supply of the snacks of their choice at the end of the study. Compliance was measured by weighing the returned serving bags and by 3DDR. 

### 2.4. Dietary Assessment

A three-day weighed diet record of all foods and beverages consumed both in and out of the home was collected from participants at baseline and during the intervention using dietary scales (Salter Electronic, Salter House wares Ltd., Kent, UK), accurate to within ±1 gram. The nonconsecutive three days included two weekdays and one weekend day over a one-week period.

The initial 3DDR was issued during the diet record instruction session at the first visit. Detailed instructions on how to collect diet records were verbally presented to each participant by a trained researcher. Written instructions were also included in the 3DDR. Pictures of different portion sizes of common food were given to the participants to help them estimate quantities when they did not prepare the meal themselves. A reminder email and text were sent the day prior to every dietary collection week to improve compliance. Participants were asked to complete a 3DDR at baseline and during the intervention, and all 3DDRs were reviewed by the researcher upon return for completeness and accuracy.

All diet records were analysed to provide an estimate of average energy and nutrient intakes using the computer programme Diet Cruncher for the PC [51]. The programme utilises food composition data from NZ Composition Database [52]. All diet records were entered by a single trained researcher to ensure consistency in data-entry decisions when substitutions had to be made. 

### 2.5. Appetite-Rating Questionnaire

Participants in the intervention groups (hazelnut, chocolate, and potato crisp groups) were also asked to record their appetite ratings on a 100 mm visual analogue scale (VAS) immediately before and after they consumed the study snacks on the same three days that dietary intake was recorded. Mean scores were calculated for the 3-day period, using the mean score from one or two days where there was missing data. The appetite-rating questionnaires included questions on hunger, desire to eat, prospective consumption, fullness, and preoccupation with thoughts of food. Hunger was assessed with the question “How hungry do you feel right now?” preceded with a 100 mm VAS, anchored with “Not at all hungry” on the left side (0 mm) to “Extremely hungry” on the right side of the scale (100 mm). Desire to eat was assessed with the question, “How strong is your desire to eat right now?” and anchored with “Strong desire not to eat” and “Strong desire to eat.” Prospective consumption was assessed with the question, “How much food could you eat right now?” and anchored with “Nothing at all” and “The most that I have ever eaten.” Fullness was assessed with the question, “How full do you feel right now?” and anchored with “Not at all full” and “Extremely full.” Preoccupation with thoughts of food was assessed with the question “Do you have any preoccupation with thoughts of food right now?” and anchored with “No thoughts of food” and “Very preoccupied, difficult to concentrate”. 

### 2.6. Physical Activity Assessment

Physical activity may influence the primary outcome measures of interest in this study, namely, body weight and composition and blood lipids and lipoproteins. Therefore, it was important to measure habitual physical activity. Physical activity was measured using NL-1000 accelerometers (New Lifestyles Inc., USA) at baseline and during the intervention. Participants were asked to wear the accelerometer clipped to their waist for a period of seven days. Detailed instructions on how to wear the accelerometer were given to the participants. The accelerometer was sealed so that participants were blinded to the activity reading. After the accelerometer was returned, information including number of steps and duration of activity was retrieved from the 7-day memory and recorded in an Excel spreadsheet. 

### 2.7. Resting Metabolic Rate

Resting metabolic rate was measured by indirect calorimetry after an overnight fast of at least 12 hours. Due to time constraints, RMR was assessed on around half of the study participants (*n*  =  52) who were randomly chosen. After a 15-minute rest period, expired gas collection was achieved through a mouthpiece with the nose clipped for a 15-minute period. Participants were asked to consume their normal diet and refrain from exercise in the 24-hour period prior to the test. They were also asked to avoid alcohol, caffeine, or nicotine within 12 hours of the test. Menstruating females were measured during the follicular phase of the menstrual cycle, as metabolic rate could be affected by the thermic effect of progesterone during the luteal phase [53]. 

### 2.8. Biochemical Indices

Venous blood samples were taken by a nurse at the Human Nutrition clinic on six occasions; two samples at baseline, twice after six weeks, and twice at the end of the study. Two blood samples on nonconsecutive days were collected during each testing week to account for intra-individual variation in blood lipid measures. Fasting blood tests were collected from participants following a 12-hour overnight fast. Ten millilitres of venous blood was collected into Vacutainers (Belton Dickinson Diagnostics) containing disodium EDTA for the analysis of plasma blood lipids and lipoproteins concentrations. Vacutainers were inverted and stored in a chilly bin containing chilled ice-pads after blood samples were drawn. All blood specimens were separated by centrifugation at 3000 g for 15 minutes at 4°C within two hours of being drawn. Once plasma and red blood cells were separated, aliquots were stored at −80°C until analysis.

Plasma total cholesterol (TC), high-density lipoprotein cholesterol (HDL-C), and triglyceride concentrations were measured in all blood samples by enzymatic methods using kits and calibrators supplied by Roche Diagnostics (Mannheim, Germany) on a Cobas Mira Plus Analyser. High-density lipoprotein cholesterol was measured in the supernatant following precipitation of apoprotein B containing lipoproteins with phosphotungstate-magnesium chloride solution [54]. Plasma low-density lipoprotein cholesterol (LDL-C) concentration was calculated using the Friedewald formula [55]. 

Calibration and quality control is maintained by participation in the Royal Australasian College of Pathologists Quality Assurance Programme. The mean intra-assay and inter-assay coefficients of variation for plasma TC, HDL-C and triglyceride were 1.12%, 4.74%, 0.73% and 2.76%, 6.91%, 2.08%, respectively. 

### 2.9. Anthropometric Measurements

Standing height was measured at baseline to the nearest millimeter using a stadiometer. Participants were asked to stand with shoes off, and their backs and heels against the back of the stadiometer. They were instructed to take a deep breath and the adjustable lever was then lowered until it was resting on the top of their head.

Body weight was measured in the morning in the fasting state during all clinic visits. Participants were weighed in light clothing without footwear, on a bioelectrical impedance analyser placed on a hard flat surface that measured to the nearest 0.01 kg. The same machine was used throughout the study. Body composition including fat mass, body fat percent, and waist fat percent was measured at baseline and at the end of the study by using dual energy X-ray absorptiometry (DXA). 

### 2.10. Statistical Analysis

In order to have 80% power to detect a difference of 0.46 kg or more in weight gain over the 12 weeks (equivalent to an annual weight change of 2.0 kg) between any two groups and assuming a standard deviation (SD) in weight change of around 0.6 kg for the 12-week period (estimated from an overfeeding study by Diaz et al. [56]) using a two-sided test with the level of significance set to 5%, 27 participants would be required in each group at the end of the study. This detectable effect size was equivalent to roughly 20% of the expected weight gain for the non-control groups based purely on the additional calories consumed with full compliance and assuming no compensatory changes in energy intake. This sample size would also be sufficient to detect effect sizes of 0.8 SD or larger in the appetite ratings in the same way. Allowing for up to 10% attrition and unusable data, 30 participants should be enrolled in each group, 120 participants in total for the four groups. 

Baseline characteristics of the participants were presented as arithmetic or geometric means and arithmetic or geometric standard deviations as appropriate. Categorical data were presented as frequencies and percentages. The effects of the four interventions on all outcomes including body weight and composition, blood lipids, RMR, appetite indices, dietary intakes, and physical activity level were examined using either regression models controlling for baseline values where measurements were available only at baseline and follow-up (body composition, RMR, appetite indices, dietary intakes and physical activity level) or linear mixed models with a random subject effect where interim measurements were also available (body weight and blood lipids). Changes within groups were shown along with their associated standard errors. Where the overall test for difference in changes between groups was statistically significant, pairwise comparisons between groups were performed. Log-transformations were used for both final and baseline (and interim where appropriate) values of dependent variables where this improved residual normality and/or homoscedasticity. 

The primary analysis was intention-to-treat (ITT) analysis, which included data from all participants who underwent randomisation. A secondary per-protocol (PP) analysis was also performed, using only participants who had at least 70% compliance to the snacks, which would be equivalent to a 2 kg weight gain over the twelve weeks without energy compensation. Stata 11.1 (StataCorp, College Station, Tex, USA) was used for all statistical analyses. All tests were performed at the two-sided 0.05 level. 

## 3. Results

**Figure 1:**
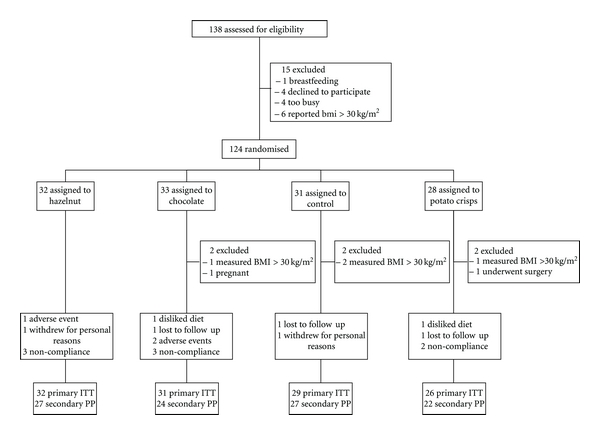
Flowchart of study participants.

Of the 124 participants who were enrolled and randomised into the study, four participants were retrospectively excluded on their first visit as their BMI was >30 kg/m^2^ despite their self-reported BMI being <30 kg/m^2^, one participant had to undergo surgery, and another one was pregnant and thus both were retrospectively excluded following their second visits. Hence, 118 participants were included in the ITT analysis: 32 participants from the hazelnut group, 31 from the chocolate group, 29 from the control group, and 26 from the potato crisp group. Of these, one participant from the chocolate group and one from the potato crisp group withdrew from the study due to their dissatisfaction with group assignment, three participants were lost to follow-up and consequently dropped out from the study, two participants withdrew from the study due to personal issues unrelated to the study, and one participant from the hazelnut group and two participants from the chocolate group with no previously noted sensitivity to these foods experienced adverse events after consuming the study snacks and were discontinued from the study (Figure 1). A similar percentage of participants from each intervention group consumed <70% of study snacks (9% in the hazelnut group, 10% in the chocolate group, and 8% in the potato crisp group). Using this criterion, 100 participants were included in the PP analysis. 

As shown in Table 1, the groups were well balanced with respect to their baseline characteristics. Among the 118 participants who were randomised into the study, 53% were females (*n*  =  63). Participants ranged in age from 18 to 65 years with a mean (SD) age of 37.4 (14.0) years. The mean (SD) height at baseline was 171.0 (9.4) cm, mean (SD) weight was 69.5 (11.4) kg, and mean (SD) measured BMI was 23.8 (3.0) kg/m^2^. 

The energy and nutrient intakes at baseline and changes from baseline to week 12 for each group are presented in Table 2. There were statistically significant differences in the percentage of energy from total fat, saturated fatty acids (SAFAs), monounsaturated fatty acids (MUFAs), polyun-saturated fatty acids (PUFAs), carbohydrate and also in the intake of vitamin E. The results from pairwise comparison showed that the percentage of total energy derived from SAFA (all *P* ≤ 0.045) and carbohydrate (all *P* ≤ 0.006) in the hazelnut group was significantly lower than all the other groups. On the other hand, vitamin E intake (all *P* ≤ 0.007), the percentage of energy derived from total fat (all *P* ≤ 0.011), MUFA (all *P* ≤ 0.001), and PUFA (all *P* ≤ 0.011) increased at 12 weeks in the hazelnut group compared to all other groups, with the exception of one pairwise comparison (hazelnut versus control; *P* = 0.057) for PUFA. Very similar results were obtained from the PP analysis.

Although there was an apparent decrease in physical activity level in the control group, there were no statistically significant differences in the changes of the anthropometric measurements, RMR, and physical activity level from baseline to 12 weeks between the groups (all *P* ≥ 0.106, Table 3). 

There was, however, a significant interaction between group and baseline BMI for waist circumference using PP analysis (*P* = 0.032). Those with higher BMI reduced their waist circumference in the nut group (*P* = 0.005) and (to a lesser extent) in the potato crisp group (*P* = 0.032) compared to the control group where the association was in the opposite direction.

Compared with baseline, the changes in plasma lipids and lipoproteins at the end of the study did not statistically significantly differ between the groups (all *P* ≥ 0.136, Table 4). 

However, in a PP analysis including only people who had >70% compliance to the study snacks, there was evidence of a difference in changes in plasma total cholesterol from baseline to week 12 between the groups (*P* = 0.035). Plasma total cholesterol in the hazelnut group was lower compared to the chocolate group (*P* = 0.006), with a tendency for the hazelnut group to be lower than the control (*P*  = 0.057). There was an additional tendency for total cholesterol in the chocolate group to be higher than the potato crisp group (*P* = 0.099). 

From the ITT analysis, there was no evidence of a difference in changes in the recordings of subjective appetite sensations among the intervention groups (all *P* ≥ 0.384, Table 5). Overall there was an increase in fullness ratings, decrease in hunger, desire to eat, prospective consumption, and preoccupation with thoughts of food ratings after consuming the study snacks. Similar results were obtained from the PP analysis. 

## 4. Discussion

The regular consumption of nuts is recommended in many national dietary guidelines. One concern with this recommendation is that, because nuts are high in fat and thus energy-dense, frequent consumption may lead to weight gain. However, epidemiological studies report that nut consumers tend to be leaner than those who do not consume nuts and clinical trials show lower than predicted weight gain from the addition of nuts. The potential mechanisms can be summarised into two routes [34–37]. Firstly, decreased energy intake via increased satiety levels and food displacements [28, 30–33, 38–44], and energy malabsorption as a result of a reduction in bioaccessibility of the fat [32, 40, 47–50], or, secondly, increased energy expenditure via increased diet-induced thermogenesis or increased RMR due to the high unsaturated fat content [30, 41, 45, 46]. It is, therefore, plausible that these unique properties of nuts help maintain energy balance. However, there is limited data comparing the body composition of those regularly consuming nuts compared to the intake of other snacks of equal energy density. It was hypothesised that consuming nuts may provide some protection against weight gain compared to other energy-dense snacks based on the aforementioned mechanisms. However, the present study found no evidence that changes in body weight or composition differed between the control group and those offered regular consumption of any of the three energy-dense snacks. In addition, blood lipid and lipoprotein response to the different snack foods did not differ significantly. Nevertheless, the diet quality among the nut consumers was appreciably improved compared to the other groups.

It was somewhat unexpected that changes in body weight and composition were not different amongst the four groups. We hypothesised that the nut group would gain less weight than predicted and that body weight would be lower compared to the other snack groups. Our study shows that the reported satiety levels after consuming the study snacks were found to be similar across all intervention groups. It appears that participants tended to compensate to a similar extent irrespective of the snack they were provided with, where 61% of the extra energy from the study snacks was displaced by reductions in other foods. This finding is in line with a recent review, which reports that the dietary compensation accounts for 55–75% of the energy from nuts [35]. No change in physical activity level was observed in the intervention groups throughout the study, and the predicted weight gain given the additional calories provided by the snacks was 2.8 kg. However, on average actual weight gain was only 0.64 kg, which equated to 23% of that predicted and did not differ between the snack groups and the control group. This compensatory response has been seen in other studies where nuts have been provided as additional foods and the observed weight gain ranged from 0 to 28% of the predicted weight gain [30–33].

One recent study comparing the effects of the consumption of almonds and cereal bars with the control group on body weight reported similar results to the current study [57]. The addition of either almonds or cereal bars did not result in a significant increase in body weight from baseline indicating a compensatory response for both foods. However, this study had a small sample size (*n*  =  45) and the energy provided from the almonds (1430 kJ) was significantly higher than that from the cereal bars (950 kJ). This discrepancy in energy makes comparison difficult but would suggest that compensation was greater in the almond group.

As with our study, research showing some degree of compensation has been conducted in non-obese populations [30, 32, 41, 45]. It is speculated that obese individuals compensate differently than their lean counterparts [41, 58, 59]. Therefore it would be interesting to repeat this study in an obese population. We note that employing a PP analysis (using 70% compliance as an indicator of adherence to the dietary advice to eat the snacks) showed that a higher baseline BMI was associated with a lower waist circumference at follow-up in the nut group compared to the control group (*P* = 0.005) and to a lesser extent in the potato crisp group compared to the control group (*P* = 0.034). Given this, dietary compensation in response to nuts and possibly potato crisps may be more pronounced in overweight individuals compared to those who are of normal weight. However, this result was only marginally statistically significant and should be interpreted with caution unless it can be replicated in other studies.

One purported mechanism whereby nuts may provide a beneficial effect on energy regulation is via an increase in energy expenditure. A previous review suggested this may account for approximately 10% of the energy contributed by nuts [36]. In the present study, RMR was measured in a subsample (*n * =  52) and there was no evidence of a difference in RMR between any of the snack groups. Previous research is somewhat mixed. Three studies showed that there was a significant increase in RMR following 2–19 weeks of peanut consumption in lean [30, 45] and overweight participants [41]. In contrast, daily almond supplementation for ten weeks [32] and six months [31] failed to show any changes in RMR or respiratory quotient. It is unclear whether the increment in RMR is specific to peanut consumption only. Given the inconsistencies among studies, this is an area requiring further research.

Another explanation provided by some researchers for the less than predicted weight gain when consuming nuts involves the reduction in the bioaccessibility of the lipid in nuts. A recent review estimates 10–15% of the energy contributed by nuts is offset by faecal loss [36]. Similar results were obtained in the first and only trial specifically designed to investigate this effect, whereby 5% of dietary fat was excreted in the whole peanut group (70 g/d), which could potentially offset around 9-10% of the energy provided by nuts [50]. However, recent studies reported that there were no statistically significant differences in body weight after consuming three different forms of hazelnuts (30 g/d) [25] and five different forms of peanuts (56 g/d) [22] for four weeks each, suggesting that the bioaccessibility of lipids was similar for all forms of nuts. Thus, the potential difference in bioaccessibility for the amount of nuts (42 g) provided in the present study may be too small to significantly influence body weight.

Changes in blood lipid and lipoprotein concentrations did not differ between the four groups. Most previous literature suggests that regular nut consumption in hypercholesterolemic individuals results in significant reductions in TC and LDL-C [21, 25, 60–64] with some showing increases in HDL-C [25, 62, 63] whilst others do not [21, 60, 61, 64]. It is likely that we did not observe an improvement in blood lipoproteins with regular nut consumption due to the low baseline TC (4.8 mmol/L) and LDL-C (2.9 mmol/L) and relatively high HDL-C (1.3 mmol/L) concentrations of this study population. Recent studies have shown that the magnitude in the reduction in TC and LDL-C following regular nut consumption was dependent on the baseline concentrations [11, 22, 65, 66]. Using PP analysis, plasma TC was significantly lower in the hazelnut group compared to the chocolate group (*P* = 0.006), with a tendency to be lower when compared to the control (*P* = 0.057). In addition, there was a tendency for the chocolate group to have higher TC than the potato crisp group (*P* = 0.099). This would indicate that among those actually following the advice to consume the different snacks, nuts show a more favourable effect on blood lipids. The cholesterol-lowering properties of nuts are largely due to their unsaturated fat content, but also due to other bioactive compounds such as phytosterols [1–5].

One important finding of this study is that the regular consumption of nuts improved diet quality compared to the consumption of other energy-dense snacks. This was particularly evident for dietary fat. The percent of energy derived from SAFA was significantly lower while the energy from MUFA and PUFA was significantly raised in the nut group compared to all the other groups. In addition, the intake of vitamin E during the intervention was significantly raised in the nut group compared to the other groups. These dietary changes support the findings of other studies, which have observed improvements in diet quality with the simple addition of nuts without any further healthy eating advice [2, 19, 38, 67]. Such changes would be expected to reduce the risk of chronic disease, in particular, CVD. A recent study has shown that substituting one unhealthy snack such as crisps and chocolate bars with one healthy snack such as unsalted nuts or seeds per day has a positive impact on nutrient density and could prevent approximately 6000 cardiovascular deaths every year in the UK [68]. 

## 5. Conclusions

Although nuts provided no additional benefits compared to isocaloric quantities of other energy-dense snacks in terms of body weight and composition, blood lipids and lipoproteins in this group of non-obese, normocholesterolaemic individuals, diet quality was substantially enhanced in the nut group. This study supports the findings of other studies, which suggest that nuts can be incorporated into the diet without the risk of adverse weight gain and can improve diet quality. 

##  Authors' Contribution

The authors' responsibilities were as follows S. L. Tey: study coordinator, designing the study, collecting, entering and analyzing the data, disseminating findings, and preparing the paper; R. Brown: study design, supervision of data collecting, data analysis, preparing the paper; A. Gray: assistance with study design, statistical analysis, editing of the paper; A. Chisholm: study design, supervision of data collection, editing of the paper; C. Delahunty: assistance with study design, editing of the paper. 

##  Conflict of Interests

None of the authors had any personal or financial conflict of interests. 

## Figures and Tables

**Table 1:** Subjects' characteristics for the total, hazelnut group, chocolate group, control group, and potato crisp group at baseline.

Variable	Total *n* = 118 mean (SD)	Hazelnut group *n* = 32 mean (SD)	Chocolate group *n* = 31 mean (SD)	Control group *n* = 29 mean (SD)	Potato crisp group *n* = 26 mean (SD)
Age (years)	37.4 (14.0)	38.9 (14.3)	38.2 (13.9)	36.1 (15.2)	35.9 (12.8)
Height (cm)	171.0 (9.4)	170.8 (8.9)	171.0 (10.4)	171.7 (9.6)	170.3 (9.2)
Weight (kg)	69.5 (11.4)	72.0 (11.1)	69.2 (13.0)	67.3 (9.5)	69.5 (11.6)
BMI (kg/m^2^)	23.8 (3.0)	24.6 (2.8)	23.6 (3.3)	22.9 (2.8)	23.9 (3.0)
Body fat (%)	26.9 (10.2)	28.1 (10.3)	26.7 (9.5)	25.8 (9.9)	26.9 (11.4)
Waist circumference (cm)	80.7 (9.4)	82.1 (8.5)	80.2 (9.6)	79.0 (8.8)	81.7 (11.1)
Women, no. (%)	63 (53%)	17 (53%)	16 (52%)	17 (59%)	13 (50%)

**Table 2:** Energy and nutrient intake at baseline and changes in intakes from baseline to week 12 for each group.

Variable	Baseline (*n* = 113) mean (SD)	Change in hazelnut (*n* = 29) mean (SE)	Change in chocolate (*n* = 26) mean (SE)	Change in control (*n* = 25) mean (SE)	Change in potato crisp (*n* = 23) mean (SE)	Overall *P* value*
Energy (kJ)^*∧*^	8669.81 (1.35)	1.06 (1.06)	0.98 (1.05)	0.96 (1.05)	1.07 (1.04)	0.721
Total fat (g)	80.12 (27.95)	22.52 (5.61)	4.85 (7.40)	−2.87 (6.90)	3.74 (3.60)	0.077
% of TE	32.56 (6.69)	6.36 (1.00)	1.27 (1.32)	−0.10 (1.46)	−0.35 (1.28)	< 0.001
SAFA (g)	31.68 (12.27)	1.38 (2.39)	4.29 (3.85)	1.58 (2.85)	1.72 (1.93)	0.620
% of TE	12.83 (3.27)	−0.37 (0.65)	1.63 (0.90)	0.89 (0.66)	0.01 (0.75)	0.005
MUFA (g)^*∧*^	24.72 (1.90)	1.67 (1.07)	1.01 (1.08)	0.95 (1.08)	1.09 (1.06)	< 0.001
% of TE	11.22 (3.14)	5.86 (0.54)	0.38 (0.61)	−0.34 (0.60)	−0.01 (0.60)	< 0.001
PUFA (g)^*∧*^	10.98 (1.71)	1.23 (1.08)	0.90 (1.09)	0.89 (1.08)	1.09 (1.07)	0.024
% of TE^*∧*^	4.68 (1.48)	1.16 (1.06)	0.90 (1.08)	0.92 (1.06)	1.03 (1.07)	0.012
Protein (g)^*∧*^	82.22 (1.43)	1.04 (1.07)	0.93 (1.06)	1.01 (1.07)	1.04 (1.06)	0.482
% of TE	16.49 (3.44)	−0.41 (0.56)	−0.99 (0.70)	0.59 (0.80)	−0.26 (0.86)	0.537
CHO (g)^*∧*^	252.16 (1.37)	0.93 (1.06)	0.97 (1.05)	0.96 (1.06)	1.08 (1.04)	0.509
% of TE^*∧*^	46.54 (1.17)	0.89 (1.03)	0.98 (1.03)	1.00 (1.03)	1.01 (1.02)	0.002
Dietary fibre (g)^*∧*^	24.69 (1.46)	0.98 (1.08)	0.90 (1.06)	1.02 (1.07)	0.95 (1.05)	0.276
Cholesterol (mg)	253.04 (127.85)	45.38 (26.53)	−4.88 (36.35)	−23.62 (31.81)	16.52 (45.73)	0.794
Sodium (mg)^*∧*^	2497.37 (1.46)	1.00 (1.11)	1.00 (1.07)	1.12 (1.11)	1.11 (1.08)	0.672
Vitamin E (mg)^*∧*^	9.13 (1.51)	1.60 (1.07)	0.90 (1.06)	0.94 (1.09)	1.30 (1.07)	< 0.001

TE: total energy; SAFA: saturated fatty acids; MUFA: monounsaturated fatty acids; PUFA: polyunsaturated fatty acids; CHO: carbohydrate.

*Overall *P*-values adjust for baseline value, sex, baseline age, and baseline BMI.

^*∧*^Geometric mean, accompanied by ratio of geometric mean.

**Table 3:** Anthropometric measurements, resting metabolic rate and physical activity level at baseline and changes in these measurements from baseline to Week 12 for each group.

Variable	Baseline mean (SD)	Change in hazelnut mean (SE)	Change in chocolate mean (SE)	Change in control mean (SE)	Change in potato crisp mean (SE)	Overall *P* value*
*Anthropometry*	(*n *= 118)	(*n *= 32)	(*n *= 29)	(*n *= 27)	(*n *= 25)	
Body weight^*Υ*^	69.55 (11.37)	0.83 (0.23)	0.59 (0.43)	0.46 (0.26)	0.50 (0.31)	0.655
BMI (kg/m^2^)	23.76 (2.99)	0.28 (0.08)	0.21 (0.14)	0.14 (0.08)	0.15 (0.11)	0.725
Fat mass (kg)^∞^	18.77 (7.92)	−0.23 (0.28)	−0.42 (0.35)	−0.29 (0.27)	−0.53 (0.30)	0.889
Body fat (%)^∞^	26.89 (10.17)	−0.75 (0.32)	−1.23 (0.39)	−0.84 (0.33)	−1.09 (0.35)	0.713
Waist fat (%)^∞^	29.62 (11.08)	1.51 (0.52)	0.86 (0.64)	0.67 (0.54)	0.76 (0.69)	0.800
Waist circumference (cm)	80.74 (9.45)	2.13 (0.89)	1.30 (0.64)	1.36 (0.62)	−0.53 (0.71)	0.106
*RMR*	(*n *= 52)	(*n *= 14)	(*n *= 12)	(*n *= 13)	(*n *= 10)	
RMR (kcal/day)	1489.29 (275.82)	−56.86 (58.40)	−69.92 (89.94)	−79.62 (38.39)	11.70 (69.37)	0.922
RQ^*∧*^	0.86 (1.08)	1.01 (1.02)	1.03 (1.04)	1.00 (1.02)	1.01 (1.02)	0.876
*PA level*	(*n *= 114)	(*n *= 29)	(*n *= 29)	(*n *= 28)	(*n *= 22)	
No. of steps/day	9215 (3691)	152 (515)	−260 (685)	−1205 (576)	190 (481)	0.217
Duration (mins)^*∧*^	28 (16)	9 (10)	9 (10)	8 (10)	9 (10)	0.668

BMI: body mass index; RMR: resting metabolic rate; RQ: respiratory quotient; PA: physical activity; no.: number.

*Overall *P*-values adjust for baseline value, sex, baseline age, and baseline BMI.

^*∧*^Geometric mean, accompanied by ratio of geometric mean.

^*Υ*^Body weight was measured with the use of bioelectrical impedance analyser.

^∞^Fat mass, percent body fat and waist fat were measured with the use of dual-energy X-ray absorptiometry.

**Table 4:** Blood lipid profile and changes in the biochemical indices from baseline to Week 12 for each group.

Variable	Baseline mean (SD)	Change in hazelnut mean (SE)	Change in chocolate mean (SE)	Change in control mean (SE)	Change in potato crisp mean (SE)	Overall *P* value*
	(*n* = 118)	(*n* = 32)	(*n* = 29)	(*n* = 27)	(*n* = 25)	
TC (mmol/L)	4.79 (0.95)	−0.06 (0.07)	0.22 (0.11)	0.10 (0.07)	0.05 (0.08)	0.211
LDL-C (mmol/L)	2.94 (0.84)	−0.09 (0.06)	0.13 (0.09)	0.09 (0.07)	−0.06 (0.07)	0.231
HDL-C (mmol/L)^*∧*^	1.32 (1.30)	1.02 (1.02)	1.04 (1.03)	1.00 (1.02)	1.04 (1.02)	0.385
TC:HDL-C ratio^*∧*^	3.57 (1.34)	0.97 (1.02)	1.01 (1.03)	1.02 (1.02)	0.97 (1.02)	0.136
TAG (mmol/L)^*∧*^	0.98 (1.48)	0.99 (1.04)	1.05 (1.04)	1.03 (1.05)	1.04 (1.05)	0.600

TC: total cholesterol; LDL-C: low-density lipoprotein cholesterol; HDL-C: high-density lipoprotein cholesterol; TAG: triglyceride.

*Overall *P*-values adjust for baseline value, sex, baseline age, and baseline BMI.

^*∧*^Geometric mean, accompanied by ratio of geometric mean.

**Table 5:** Changes in appetite indices before and after consuming study snacks during the intervention.

Variable	Before consumption mean (SD)	Change in hazelnut mean (SE)	Change in chocolate mean (SE)	Change in potato crisp mean (SE)	Overall *P* value*
	(*n* = 89)	(*n* = 32)	(*n* = 31)	(*n* = 26)	
Hunger (mm)	56.33 (19.66)	−25.67 (5.16)	−13.37 (5.10)	−19.10 (3.34)	0.384
Desire to eat (mm)	59.39 (20.64)	−25.33 (4.68)	−22.05 (4.72)	−20.66 (3.10)	0.874
Prospective consumption (mm)	50.64 (17.89)	−17.81 (3.76)	−12.31 (3.97)	−12.50 (2.52)	0.921
Fullness (mm)	41.96 (17.68)	17.99 (4.91)	10.59 (4.20)	10.74 (3.33)	0.631
Preoccupation with thoughts of food (mm)	51.80 (19.20)	−22.49 (4.25)	−15.57 (4.58)	−18.61 (3.29)	0.760

*Overall *P*  values adjust for baseline value, sex, baseline age, and baseline BMI.
